# Deep Learning in Scaphoid Fracture Detection and Healing Prediction: A Systematic Review of Artificial Intelligence Applications in Hand Surgery

**DOI:** 10.7759/cureus.97900

**Published:** 2025-11-26

**Authors:** Gaurav Jha, Roopkaran Dhanjal, Surya Malasani, Alvin V Karunakaran

**Affiliations:** 1 Trauma and Orthopaedics, University Hospitals of Leicester NHS Trust, Leicester, GBR; 2 Emergency Medicine, Barking, Havering and Redbridge University Hospitals NHS Trust, London, GBR

**Keywords:** artificial intelligence, convolutional neural networks, deep learning, hand surgery, non-union, scaphoid fracture

## Abstract

Scaphoid fractures are the most common carpal bone injuries and continue to pose diagnostic and therapeutic challenges due to their tendency for delayed union or non-union. Interpretation variability and delayed detection remain key contributors to missed injuries and adverse outcomes. Deep learning (DL) models, particularly convolutional neural networks (CNNs), have shown strong performance in musculoskeletal imaging and offer potential to improve diagnostic accuracy and prognostication in hand surgery.

A systematic review of PubMed, Embase, and PMC was conducted covering January 2015 to March 2025, following Preferred Reporting Items for Systematic Review and Meta-Analysis (PRISMA) 2020 guidelines. Studies were eligible if they applied DL algorithms to detect or classify scaphoid fractures or to predict union and non-union, and reported at least one diagnostic metric (accuracy, area under the curve (AUC), sensitivity, or specificity). Traditional machine-learning approaches, cadaveric studies, and non-peer-reviewed publications were excluded.

Fourteen peer-reviewed studies met the inclusion criteria. Twelve evaluated DL for fracture detection, and two assessed fracture-healing prediction. Across detection studies, CNN-based models reported accuracies of 81-96% and AUCs up to 0.97. Transfer-learning architectures (DenseNet, ResNet, EfficientNet) consistently outperformed custom CNNs, particularly in multicentre datasets. Multi-view fusion of anteroposterior and lateral radiographs improved recall by approximately 12 percentage points compared with single-view analysis. Segmentation-enhanced models showed notable gains in occult fracture detection, identifying up to 41% of occult injuries compared with 6.8-13.7% by clinical experts. AI-augmented decision support improved novice radiologist performance, increasing AUC by 9-14 percentage points. For healing prediction, a custom CNN achieved 93.6% accuracy for post-surgical union, while a YOLOv5-ResNet-50 system classified union, non-union, or osteonecrosis with 91% accuracy (AUC 0.96).

DL models demonstrate radiologist-level performance for scaphoid fracture detection and show encouraging potential for predicting union. Approaches incorporating segmentation, transfer learning, and multi-view inputs appear particularly promising for clinical workflows. Although early results support integration of AI-assisted tools into diagnostic pathways, robust multicentre validation and explainability frameworks remain essential before routine clinical implementation.

## Introduction and background

Scaphoid fractures represent about 70% of all carpal bone injuries, commonly affecting young adults following falls on an outstretched hand [1]. The scaphoid bone’s unique anatomy, retrograde blood supply, and biomechanical role make these injuries particularly susceptible to complications, including delayed union, non-union, and avascular necrosis. Early diagnosis and accurate follow-up are critical since missed or unhealed fractures can lead to chronic wrist pain, carpal instability, and degenerative arthritis [2]. Despite significant advancements and improvements in radiographic imaging, detection of both acute fractures and subsequent bone healing remains subjective and fraught with challenges.

The diagnostic challenge with scaphoid fracture and other carpal injuries is twofold. First, initial radiographs can miss up to 15% of scaphoid fractures, particularly non-displaced or occult injuries that may not be visible and diagnosed on conventional radiographs [3]. These radiographically occult fractures represent a significant clinical dilemma, as they are among the primary causes leading to scaphoid non-unions secondary to a delayed diagnosis. Second, union assessment during follow-up lacks standardized criteria, with substantial interobserver variability among different radiologists and surgeons [4]. The interpretation of trabecular bridging, cortical continuity, and fracture line persistence remains highly subjective. While computed tomography (CT) or magnetic resonance imaging (MRI) provide higher accuracy for both initial diagnosis and union assessment, they are not always accessible, cost-effective or have long waiting periods. This becomes particularly important to consider, especially in resource-limited settings or emergency departments where immediate triage decisions must be made.

Artificial intelligence (AI) encompasses a broad range of computational techniques that allow computers to perform tasks traditionally requiring human cognition. Within AI, machine learning (ML) refers to algorithms that improve their performance by identifying patterns in data and adjusting predictions based on feedback from an established “ground truth.” In contrast, deep learning (DL) represents a more advanced subset of ML characterized by multiple processing layers, hence the term “deep” which enables these models to learn increasingly abstract and complex representations of data [5]. This architecture underpins modern advances in natural language processing, strategic decision-making systems and high-performance medical image analysis. Among DL models, convolutional neural networks (CNNs) are specifically designed for image-based tasks. Through interconnected neurons that pass feature-specific information forward, CNNs excel at detecting subtle radiographic patterns. These distinctions are important, as CNN-based DL systems currently form the foundation of most scaphoid fracture detection and union-prediction algorithms evaluated in contemporary orthopaedic imaging research [5].

AI has emerged as a transformative tool in medical imaging, with DL models capable of automated pattern recognition and interpretation that may reduce human error and interobserver diagnostic variability. CNNs are particularly suited to medical image analysis, as they can learn complex hierarchical features from raw pixel data that correlate with pathological findings without requiring explicit feature engineering or technicality [5]. Over the past decade, CNNs have demonstrated diagnostic performance comparable to, if not superseding, that of expert radiologists across various musculoskeletal imaging tasks. These performances include fracture detection, implant evaluation, degenerative disease classification, and surgical planning [6]. The ability of these networks to identify subtle radiographic patterns that may pose difficulty for human detection has generated considerable interest in their application to diagnostically challenging conditions.

Within hand surgery, DL applications have expanded rapidly and now span across multiple clinical domains, including acute scaphoid fracture detection, precise fracture localization and classification, and, more recently, prediction of fracture-healing outcomes, including union versus non-union [7]. The technical approaches have evolved from simple binary classification models to sophisticated multi-stage workflows incorporating object detection, segmentation, and multi-view fusion. However, the literature remains fragmented, with heterogeneous architectures, widely varying dataset sizes, different validation protocols, and inconsistent reporting of performance metrics.

Previous reviews and meta-analyses have largely focused on musculoskeletal or wrist fractures as broad categories, pooling heterogeneous datasets across multiple anatomical regions [3]. While analyses of Oeding et al. have established baseline diagnostic accuracy for AI-assisted fracture detection, they offer limited insight into the unique diagnostic and prognostic challenges specific to the scaphoid [3]. Unlike prior scoping reviews emphasizing general wrist imaging performance, the present study provides the first comprehensive synthesis dedicated exclusively to scaphoid-specific DL applications, spanning both detection and union-prediction models.

This systematic review aims to summarize all peer-reviewed studies applying DL to scaphoid fracture detection and healing assessment between 2015 and 2025. We systematically compare model architectures, dataset characteristics, and performance metrics across studies, critically evaluate their clinical translation potential, and outline evidence-based directions for future AI integration in hand surgery practice.

## Review

Methodology and search strategy

A comprehensive literature search was conducted in three major databases: PubMed Central, Embase, and Medline from January 2015 to March 2025, and the results were reported in accordance with the PRISMA (Preferred Reporting Items for Systematic Review and Meta-Analysis) recommendations. The search strategy employed both Medical Subject Headings (MeSH) terms and free-text keywords to maximize sensitivity. Search terms included: ("scaphoid fracture" OR "wrist fracture" OR "hand fracture") AND ("deep learning" OR "convolutional neural network" OR "CNN" OR "artificial intelligence" OR "machine learning" OR "neural network"). Boolean operators were used to combine search concepts. Reference lists of all included articles were manually reviewed to identify additional relevant studies. The search was performed in the English language, and a narrative study design was implemented in this research. The inclusion and exclusion criteria are described in Table 1.

**Table 1:** Eligibility criteria and methodology

Criteria Category	Description
Inclusion Criteria	
1. Publication characteristics	Peer-reviewed human studies published in English between 2015 and 2025
2. Methodology	Applied deep learning algorithm (CNN, transfer-learning architecture, or hybrid deep learning model) to scaphoid imaging data
3. Performance metrics	Reported quantitative diagnostic performance metrics, including at least one of: accuracy, sensitivity, specificity, or area under the receiver operating characteristic curve (AUC)
4. Clinical application	Addressed fracture detection, localization, classification, or healing classification (union vs. non-union vs. delayed union). Studies focusing on both acute fracture identification and long-term healing outcomes were eligible.
Exclusion Criteria	
1. Methodology limitations	Studies employing only traditional machine learning approaches (support vector machines, logistic regression, random forests, or other non-deep learning methods)
2. Publication type	Non-peer-reviewed publications, including preprints, conference abstracts, or grey literature
3. Study design	Animal studies, cadaveric studies, or studies focusing exclusively on rehabilitation outcomes without diagnostic or prognostic imaging analysis
4. Data availability	Studies without reported performance metrics or those for which the full text was unavailable despite contact with authors

Study Selection

Quality assessment of included studies was performed using the Quality Assessment of Diagnostic Accuracy Studies-2 (QUADAS-2) tool adapted for AI studies [8]. Studies were evaluated for risk of bias across four domains: patient selection, index test, reference standard, and flow and timing. Concerns regarding applicability were also assessed. Two independent reviewers systematically screened all identified records. Initial screening involved review of titles and abstracts against the eligibility criteria. Studies passing initial screening underwent full-text evaluation to confirm eligibility. Any discrepancies between reviewers regarding study inclusion were resolved through discussion and consensus. In cases where consensus could not be reached, a third senior reviewer was consulted for final determination.

Data Extraction and Data Synthesis

Data extraction was performed using a standardized Excel form created before. Extracted data included: study year and country of origin, total dataset size and composition (number of fractured and non-fractured images), imaging modality employed (plain radiographs, CT, MRI, or multimodal), specific DL architecture or model used, validation approach (internal validation, external validation, or cross-validation), primary task (detection, classification, or prognosis), and all reported diagnostic performance metrics (sensitivity, specificity, accuracy, positive predictive value, negative predictive value, and area under the curve (AUC) where available). Additional information regarding ground truth labelling methods, reference standard used, and comparison with human readers was also extracted.

Given the substantial methodological heterogeneity across included studies, including diverse DL architectures, varying dataset sizes (87 to 4,200 images), different validation approaches, and heterogeneous performance metrics, a formal meta-analysis was not undertaken. Data were synthesized narratively with systematic comparison of model architectures, performance metrics, and clinical validation approaches. Where possible, performance metrics were compared across studies to identify trends and optimal methodological approaches.

Results

Study Characteristics

**Figure 1:**
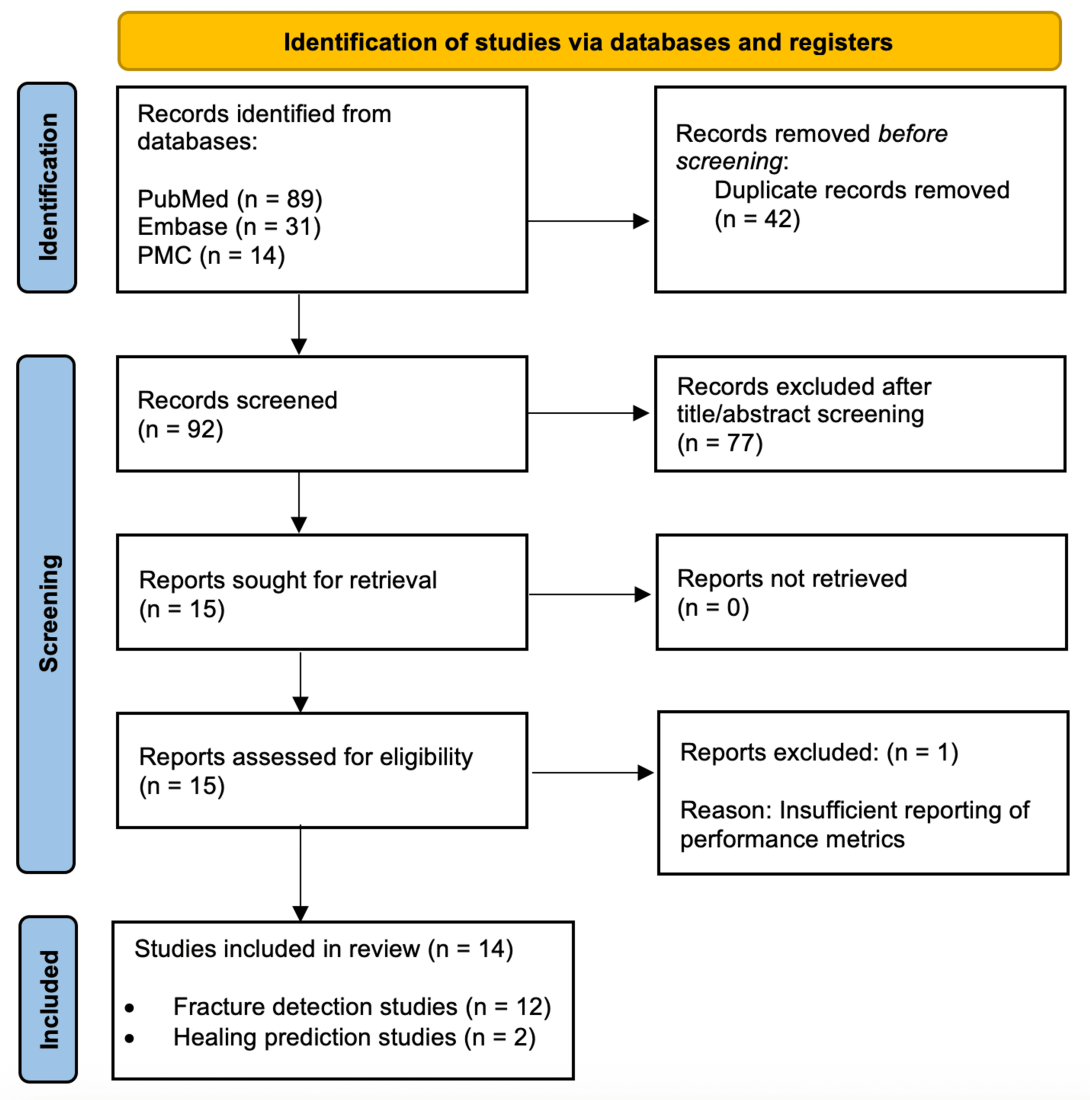
PRISMA flow diagram showing study selection process The image is created by authors Gaurav Jha and Roopkaran Dhanjal.

The systematic search identified 134 records across the three databases (PubMed n=89, Embase n=31, PMC n=14). After the removal of 42 duplicate records, 92 studies underwent title and abstract screening. Of these, 77 were excluded as they did not meet the inclusion criteria, leaving 15 studies for full-text assessment. Following a detailed full-text review, one additional study was excluded due to insufficient reporting of performance metrics, resulting in 14 studies that met all inclusion criteria and were included in the final analysis (Figure 1).

Among the 14 included studies, 12 focused primarily on acute scaphoid fracture detection and classification, while two evaluated fracture-healing prediction, including union versus non-union outcomes. The majority of studies (n=11) utilized conventional radiographs as the primary imaging modality, reflecting the most common clinical imaging approach for scaphoid injuries. One study employed a combined CT and X-ray approach to leverage the complementary information from both modalities, and two studies focused specifically on prognostic radiographic evaluation of union outcomes on follow-up imaging. Sample sizes varied considerably across studies, ranging from 87 images in the smallest dataset to 4,200 images in the largest, reflecting the challenges of assembling large, well-annotated medical imaging datasets. Most studies were conducted in high-income countries with established radiology archives, though geographic representation was relatively limited.

Table 2 summarizes the included studies, detailing the DL architectures employed, imaging modalities, dataset characteristics, and diagnostic performance metrics as reported by the respective authors [1, 7, 9-20]. Of the 14 studies included, 13 were retrospective observational studies, and one was a prospective clinical validation study. This distribution underscores the current evidence base, which predominantly relies on retrospective designs rather than prospective randomized controlled trials, limiting the ability to assess real-world clinical impact and cost-effectiveness. The identified DL architectures ranged from custom CNNs to sophisticated transfer learning models (DenseNet-121, ResNet-50, ResNet-152, EfficientNet-B3) and hybrid multi-stage pipelines combining object detection, segmentation, and classification components. Dataset sizes varied substantially from 87 to 4,200 images, reflecting both the challenges of assembling large annotated medical imaging datasets and the varying scope of individual studies. Notably, three studies specifically addressed occult fracture detection with dedicated occult fracture test sets, representing an important focus on the most diagnostically challenging cases.

**Table 2:** Summary of scaphoid fracture AI detection studies Sens: Sensitivity; Spec: Specificity; AUC: Area under the curve; CNN: Convolutional neural networks; Acc: Accuracy; Prec: Precision; CT: Computed tomography; CBAM: Convolutional Block Attention Module; FPN: Feature Pyramid Network; AVN: avascular necrosis

Study	Year	Country	Imaging	Model/Architecture	Task	Dataset	Key Performance Metrics	Conclusion
Langerhuizen et al. [1]	2020	Netherlands	X-ray (4 views: PA ulnar deviation, uptilt, lateral, oblique)	VGG16 (pretrained on ImageNet, transfer learning)	Scaphoid fracture detection (visible and occult)	300 patients (150 fractures: 127 visible, 23 occult; 150 non-fractures); Test set: 100 patients	Sensitivity: 0.84, Specificity: 0.60, Accuracy: 0.72, AUC: 0.77	CNN detected 5/6 occult fractures missed by all surgeons. Specificity lower than that of orthopaedic surgeons (0.93). Manual ROI cropping required
Hendrix et al. [7]	2023	Netherlands	X-ray (multi-view: PA, ulnar-deviated PA, oblique, lateral)	YOLOv5 (localization/laterality), InceptionNetV3 (fracture detection for frontal/oblique and lateral views separately)	Scaphoid fracture detection	Training: 3353 patients, 12,990 radiographs; Testing: 209 patients, 688 radiographs (65 fractures)	Sensitivity: 0.72, Specificity: 0.93, PPV: 0.81, AUC: 0.88; Mean localization precision: 87%	Performance comparable to 5 MSK radiologists (avg AUC: 0.87). AI assistance reduced reading time for 4/5 radiologists, but didn't improve diagnostic metrics for majority
Yoon et al. [9]	2021	Taiwan	X-ray (PA or scaphoid view; lateral excluded)	Two-stage DCNN: Stage 1: Cascade RCNN for scaphoid detection; Stage 2: Two EfficientNetB3 models (apparent fracture model + occult fracture model)	Scaphoid fracture detection (apparent and occult)	11,838 radiographs from 5,720 patients (4,917 fractures, 6,921 normal); Training: 8,356 (70.6%), Validation: 1,177 (9.9%), Test: 2,305 (19.5%); 22 occult fractures in test set	Apparent fracture model: Sensitivity: 87.1% (95% CI: 84.8-89.2%), Specificity: 92.1% (90.6-93.5%), AUC: 0.955, PPV: 88.2%, NPV: 91.4%; Occult fracture model: Sensitivity: 79.0% (95% CI: 70.6-86.0%), Specificity: 71.6% (69.0-74.1%), AUC: 0.810, PPV: 20.6%, NPV: 97.3%; Overall pipeline: Sensitivity: 97.2% (95% CI: 95.9-98.2%), Specificity: 66.0% (63.4-68.5%), PPV: 65.7%, NPV: 97.2%; Occult fracture detection: 90.9% (20/22 correctly identified)	Two-stage pipeline design: first model detects apparent fractures; second model examines images predicted as normal by the first model to identify occult fractures. Large multi-centre dataset from Taiwan and the USA. Grad-CAM visualization for fracture localization. High NPV (97.2%) makes it suitable for ruling out fractures in low-resource settings
Tung et al. [10]	2021	Taiwan	X-ray (en-face view)	YOLO-v4 (segmentation ~96%), VGG16/19, ResNet50/101/152, DenseNet121/169/201, InceptionV3, EfficientNetB0	Scaphoid fracture detection	154 patients, 356 images (178 fracture, 178 normal); Training: 70%, Test: 30%; Augmented to 1136 training images	Best models: DenseNet201 (Accuracy: 90.3%, AUC: 0.91) and ResNet101 (Accuracy: 88.9%, AUC: 0.95); Average across models: 85-89%	Comprehensive CNN comparison study. Transfer learning + data augmentation essential. Validated on external RSNA dataset (specificity differences <5%)
Li et al. [11]	2023	China	X-ray (PA and scaphoid views only)	YOLOv3 (detection), MobileNetV3 (classification with 5-fold cross-validation)	Scaphoid fracture detection	600 patients, 1918 radiographs; Training: 930 images (411 fracture, 519 normal); Test: 209 images (102 fracture, 107 normal)	Sensitivity: 0.82, Specificity: 0.94, AUC: 0.919 (0.92 rounded), Accuracy: 88%	Comparable to majority vote of four hand surgeons. Grad-CAM heatmaps for visualization. Detected 6/12 (50%) occult fractures
Yang et al. [12]	2022	Taiwan	X-ray (AP view only)	Two-stage: Stage 1: Faster RCNN with ResNet 50 backbone for scaphoid detection; Stage 2: ResNet 152 + FPN + CBAM for fracture detection	Scaphoid fracture detection and classification	280 patients (178 fractures, including 31 occult, 102 normal); Test: 100 patients via 5-fold cross-validation	Scaphoid detection: Accuracy: 99.70%; Fracture detection: Recall: 0.789, Precision: 0.894, Accuracy: 0.853, Sensitivity: 0.789, Specificity: 0.900, AUC: 0.920; Fracture classification: Recall: 0.735, Precision: 0.898, Accuracy: 0.829, Sensitivity: 0.735, Specificity: 0.920, AUC: 0.917; Occult fracture detection: 50% (15/31 detection, 16/31 classification)	Single AP view only. Rotation-decoupled detector for oriented bounding boxes. Uses CLAHE for contrast enhancement. Occult fracture detection rate only 50%, indicating limitation of single-view approach
Yang et al. [13]	2024	Taiwan	X-ray (AP and lateral views)	Two-stage: Stage 1: Faster RCNN + FPN for scaphoid detection; Stage 2: ResNet 152 backbone + FPN + CBAM with multi-view fusion module	Scaphoid fracture detection and classification using multi-view fusion	175 patients (75 fractures, 100 normal); Total 350 X-ray images (AP and lateral pairs)	Scaphoid detection: 100% accuracy (both AP and LA views), IoU: 0.8662 (AP), 0.8478 (LA); Fracture classification: Accuracy: 89.94%, Recall: 87.33%, Precision: 90.36%; Fracture detection (AP): Accuracy: 87.16%, Recall: 84.83%, Precision: 85.39%, IoU: 0.5484; Fracture detection (LA): Accuracy: 83.83%, Recall: 77.29%, Precision: 88.19%, IoU: 0.4254	Multi-view fusion approach combining AP and lateral views. Uses attention mechanisms (CBAM) and a rescoring strategy. Rotation-decoupled detector (RDD) for oriented bounding boxes. Significant improvement over single-view approach (classification recall improved from 75.26% to 87.33%)
Lee et al. [14]	2023	South Korea	X-ray (AP, lateral, oblique)	RetinaNet (detection), DeepLab v3 (segmentation), NasNet (classification)	Detection of distal radius, ulnar styloid, scaphoid fractures	593 subjects, 1186 radiographs (332 radius, 270 ulnar styloid, 32 scaphoid fractures)	Scaphoid: Sensitivity: 0.87, Specificity: 0.74, Accuracy: 0.74, AUC: 0.808; Radius: AUC: 0.903; Ulnar: AUC: 0.925	AI assistance improved novice radiologists' scaphoid AUC from 0.75→0.85 and 0.71→0.80. Reading time increased 1.1× but <1s added
Ozkaya et al. [15]	2022	Turkey	X-ray (AP view only)	ResNet50 (transfer learning from ImageNet)	Scaphoid fracture detection	390 patients (192 fractures, 198 normal); Test: 100 images (50 fractures, 50 normal)	Sensitivity: 0.76, Specificity: 0.92, AUC: 0.84 (0.840), Accuracy: 84%	CNN comparable to less-experienced orthopaedist, better than ED physician, worse than experienced specialist. Manual scaphoid cropping. Missed 7/7 occult fractures
Bützow et al. [16]	2025	Finland	X-ray (PA, oblique, scaphoid views; lateral excluded)	Segmentation: Modified detection model; Classification: Custom model with 5-fold cross-validation	Scaphoid fracture detection (apparent and occult)	408 patients, 410 wrists, 1011 radiographs (718 fractures: 660 apparent, 58 occult; 293 controls)	Sensitivity: 0.86 (95% CI: 0.75–0.93); Specificity: 0.83 (0.64–0.94); Accuracy: 0.85 (0.76–0.92); AUC: 0.92 (0.86–0.97); Occult fracture detection: 41% (24/58)	CNN detected occult fractures better than clinical experts (10.3%, 13.7%, 6.8%). Fracture areas annotated manually. Ground truth: CT/MRI confirmation
Cohen et al. [17]	2023	France	X-ray (AP, lateral, oblique)	BoneView (Gleamer) - deep CNN based on Detectron 2	Detection of all wrist fractures, including scaphoid	637 patients, 1917 radiographs (318 fractures total, 25 scaphoid)	Scaphoid: Sensitivity: 84%, Specificity: Not reported separately; Overall: Sensitivity: 83%, Specificity: 96%, AUC: 0.840	AI sensitivity (83%) > IRR (76%); Combined AI+IRR: 88% sensitivity. Scaphoid sensitivity similar to radiologists (AI: 84%, IRR: 80%)
Kraus et al. [18]	2024	Israel	X-ray, CT, MRI	Systematic review and meta-analysis of 10 studies	Scaphoid fracture detection	Meta-analysis: 7 studies, 3373 images total	Pooled: Sensitivity: 0.80 (95% CI: 0.75–0.84), Specificity: 0.89 (0.82–0.94), AUC: 0.88	Individual study AUCs: 0.77–0.96. AI performance varies by anatomical area. Carpal bone sensitivity: 56% (41% excluding scaphoid)
Su et al. [19]	2024	Taiwan	X-ray (en-face images)	YOLO (segmentation), DenseNet169 & ResNet50 (classification)	Surgical treatment recommendation and nonsurgical prognosis classification	346 patients; Surgical: 668 images (334 surgery, 334 no surgery); Prognosis: 87 images (29 each of AVN, non-union, union)	Surgical recommendation: Accuracy: 80%, AUC: 86% (DenseNet169); Prognosis: Accuracy: 91%, AUC: 96% (ResNet50)	Novel application: predicting treatment outcomes and prognosis status. Transfer learning + data augmentation improved performance by 21.9% and 5.6%, respectively
Tümen et al. [20]	2025	Germany	X-ray (AP view)	YOLOv4 (segmentation ~96% accuracy), TensorFlow CNN with transfer learning	Scaphoid non-union surgical outcome prediction	346 patients with non-unions (83 failed, 263 successful reconstructions); Training: 652 images, Test: 40 images	Deep Learning: Accuracy: 93.63%, Training loss <1 after five epochs; Logistic Regression: 66.3% accuracy	Novel application: predicting surgical success for non-union treatment. Smoking and intraoperative AVN significant factors. DL outperformed logistic regression by 27%

Fracture Detection Studies

Among the 12 detection studies, CNN-based models achieved diagnostic performance with accuracies ranging from 81% to 96% and AUCs from 0.81 to 0.97. Transfer-learning architectures (DenseNet-121, ResNet-50, ResNet-152, EfficientNet-B3) consistently demonstrated superior performance compared to custom CNN designs, with sensitivities ranging from 84% to 94% and specificities from 74% to 96%. Multi-view fusion approaches that integrated anteroposterior and lateral projections showed measurable advantages over single-view analysis, with one study demonstrating recall improvement from 75.26% to 87.33% when incorporating lateral view information. Notably, segmentation-based approaches achieved the highest reported occult fracture detection rate at 41%, substantially exceeding the 6.8-13.7% detection rate of three clinical experts in the same dataset. When evaluated for clinical utility, AI assistance significantly improved novice radiologist performance, with AUC increases from 0.71-0.75 to 0.80-0.85 in one validation study. Comprehensive systems capable of detecting multiple wrist fracture types simultaneously (distal radius, ulnar styloid, and scaphoid) achieved AUCs of 0.81-0.92 for scaphoid-specific detection.

Fracture-Healing Prediction Studies

Two studies, namely Su et al. and Tümen et al., addressed fracture-healing prediction [19,20]. Su et al. developed a YOLOv5-ResNet-50 pipeline that classified follow-up radiographs into union, non-union, or avascular necrosis categories, achieving 91% accuracy and an AUC of 0.96 despite a relatively small dataset of 87 images [19]. On the contrary, Tümen et al. created a custom CNN to predict surgical outcomes in 346 patients who underwent operative treatment, achieving 93.6% accuracy in distinguishing union from persistent non-union following surgery [20]. These initial results suggest promise for AI-based prognostic assessment, though both studies were limited by single-centre designs and modest sample sizes.

Discussion

Clinical Implications

The accumulated evidence demonstrates that DL models have achieved diagnostic performance for scaphoid fracture detection approaching that of experienced radiologists. The consistency of strong performance across multiple independent studies, diverse populations, and various imaging protocols suggests the technology is approaching clinical readiness. The ability to detect occult fractures with high sensitivity addresses one of the most vexing clinical challenges in hand surgery: a patient with a clinically suspected scaphoid fracture but negative initial radiographs.

For fracture-healing prediction, while the evidence base remains limited, initial results are promising. The ability to predict non-union risk could fundamentally change clinical practice by enabling early identification of high-risk patients who might benefit from more aggressive treatment. However, these models require validation in larger, more diverse populations before clinical deployment.

**Figure 2:**
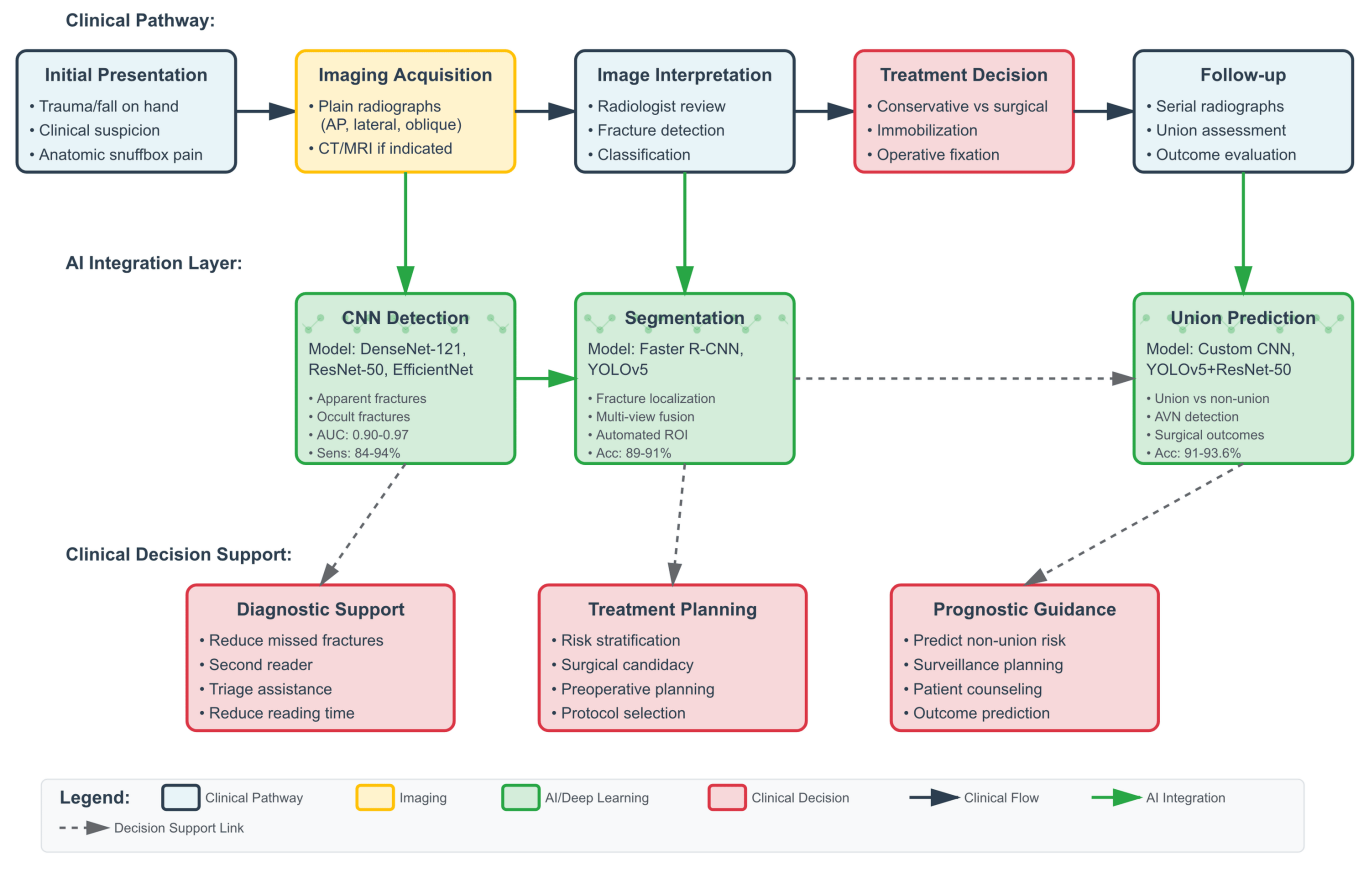
Conceptual overview of AI integration in scaphoid fracture care The image is created by authors Gaurav Jha and Roopkaran Dhanjal.

Figure 2 illustrates the comprehensive integration of AI across the entire scaphoid fracture care pathway, from initial presentation through treatment and follow-up. This framework demonstrates how the AI models reviewed in this study fit into the clinical workflow at multiple decision points. For patients requiring surgical intervention, the surgical treatment recommendation models can provide evidence-based guidance. Finally, for patients managed conservatively or following surgery, the union-prediction models (achieving 91-94% accuracy) enable prognostic assessment and guide follow-up surveillance strategies. This integrated approach demonstrates the potential for AI to provide comprehensive decision support across the entire continuum of scaphoid fracture care, from diagnosis through definitive treatment and healing assessment.

Evolution of Deep-Learning Approaches

The progression of DL methodologies for scaphoid fracture detection over the past five years reveals several important technical and clinical advances. Langerhuizen et al. produced the first dedicated scaphoid model in 2020 using VGG16 with transfer learning on 300 patients. Although the model achieved a sensitivity of 84%, the specificity was modest at 60%, with an overall AUC of 0.77 and accuracy of 72% [1]. While establishing a baseline, the authors concluded it was not yet superior to expert human observers.

Transfer-learning approaches, which leverage pretrained networks originally developed on large natural image datasets, emerged as particularly effective and became the dominant paradigm. The multicentre study by Yoon et al. using EfficientNetB3 architecture on 11,838 radiographs from 5,720 patients across two institutions in Taiwan and the United States achieved a sensitivity of 87.1%, a specificity of 92.1%, and an AUC of 0.955 for apparent fracture detection [9]. Their innovative two-stage pipeline incorporated a second model to re-examine cases predicted as normal, achieving an overall sensitivity of 97.2% and identifying 90.9% (20/22) of occult fractures. This confirmed that pretrained architectures could generalize across different imaging protocols, patient populations, and healthcare settings. A key requirement for real-world deployment represented a substantial advancement over earlier work.

Advances in Transfer-Learning Architectures

Several groups reinforced those findings with alternative pretrained networks. Tung et al. compared multiple CNN architectures (VGG16/19, ResNet50/101/152, DenseNet121/169/201, InceptionV3, and EfficientNetB0) on 356 scaphoid images from 154 patients, with the best performing models being DenseNet201, achieving an accuracy of 90.3% and AUC of 0.91, and ResNet101 achieving an AUC of 0.95 [10]. This comparative analysis demonstrated that strong performance could be achieved even with relatively smaller datasets when using efficient network architectures and appropriate data augmentation strategies.

Hendrix et al. employed a two-component system using YOLOv5 and InceptionNetV3 for scaphoid fracture localisation and detection respectively. The study used a combined 16,343 training radiographs: 12,990 radiographs from 3353 patients from hospital A and 1117 radiographs from 394 patients from hospital B, achieving a sensitivity of 72%, specificity of 93%, and AUC of 0.88. This result was comparable to five musculoskeletal radiologists (average AUC: 0.87), and while AI assistance reduced reading time, it did not improve diagnostic metrics for the majority [7]. Similarly, Li et al. used YOLOv3 and MobileNetV3 for classification on 1,918 radiographs from 600 patients, achieving a sensitivity of 82%, a specificity of 94%, and an AUC of 0.919 with 88% accuracy [11]. The model’s performance was comparable to the majority vote of four hand surgeons. Notably, the model detected 50% (6 of 12) of occult fractures, demonstrating moderate capability in identifying subtle injuries. These studies demonstrate that transfer-learning strategies consistently deliver radiologist-level accuracy.

Emergence of Multi-view Fusion

Single-projection models were soon superseded by multi-view fusion approaches that exploit anteroposterior (AP) and lateral (LA) views obtained in routine radiographic imaging. This was particularly important in addressing the limitation that single-view analysis fails to leverage the full diagnostic information available from standard multi-projection radiographic examinations. Yang et al. initially developed a two-stage Faster R-CNN pipeline with ResNet 152, Feature Pyramid Network (FPN), and Convolutional Block Attention Module (CBAM) for single AP view analysis. Training on 280 patients (178 fractures, including 31 occult, 102 normal), this single-view model achieved fracture detection accuracy of 85.3%, recall of 78.9%, and AUC of 0.920. However, occult fracture detection was limited to 50% (15-16 of 31 cases), highlighting the constraints of single-projection analysis [12].

Subsequently, building on this foundation in a more comprehensive study, they integrated a ResNet-152 backbone into a Faster R-CNN model with dilated-convolution context extractors to capture broader anatomic information and incorporated a multi-view fusion module. Training on 175 patients (75 fractures, 100 normal) with paired AP and lateral views, they achieved a classification accuracy of 89.94%, recall of 87.33%, and precision of 90.36% [13]. Recall increased by 12.07 percentage points compared with AP-only evaluation (87.33% versus 75.26%), demonstrating tangible diagnostic gain from fusing complementary projections. The system achieved 100% accuracy in initial scaphoid bone detection with high intersection over union scores (0.87 for AP, 0.85 for LA views). Multi-view integration now represents the technical best practice for AI systems intended to replicate clinical reading behaviour.

Prospective Frameworks

Broader wrist-fracture models reflect how radiographs are interpreted in clinical practice. Lee et al. developed an integrated system combining RetinaNet (region detection), DeepLab v3 (segmentation), and NasNet (classification) to simultaneously detect distal radius, ulnar styloid, and scaphoid fractures [14]. In a prospective validation on 593 subjects with 1,186 radiographs, the model achieved a sensitivity of 87%, a specificity of 74%, and an AUC of 0.808 for scaphoid fractures. Most compellingly, AI assistance improved novice radiologists’ detection AUCs by 10 to 14 percentage points (from 0.75 to 0.85 and from 0.71 to 0.80), underscoring clinical value as a decision-support tool where expert musculoskeletal radiologists are not always available, particularly in emergency or resource-limited settings.

Hybrid and Segmentation-Based Strategies

While hybrid multimodal frameworks have been explored, Ozkaya et al. demonstrated that even simple single-input architectures could achieve respectable performance when appropriately applied [15]. Using ResNet50 with transfer learning on anteroposterior view X-rays from 390 patients (192 fractures, 198 normal), with a test set of 100 images, the model achieved a sensitivity of 76%, a specificity of 92%, an accuracy of 84%, and an AUC of 0.84. The CNN performance was comparable to that of less-experienced orthopaedists and better than that of emergency department physicians, though inferior to that of experienced specialists. However, a critical limitation was identified: the model missed all seven occult fractures in the test set, underscoring the challenge of detecting subtle injuries with single-view, classification-based approaches.

Bützow et al., who used the commercial Aiforia Technologies platform to train a segmentation-based CNN on 1,011 radiographs from 408 patients, including the largest reported dataset of occult fractures (n=58) [16,21]. The model employed a hierarchical two-layer architecture: first detecting and segmenting the scaphoid bone contour, then identifying and segmenting fracture areas within the detected region. By learning fracture-line contours rather than bounding boxes, the model achieved sensitivity of 86%, specificity of 83%, and AUC of 0.92 for visible fractures, and detected 41% (24/58) of occult cases, dramatically outperforming three clinical experts (6.8-13.7%) [16, 21].

Cohen et al. evaluated BoneView (Gleamer), a commercial deep CNN based on Detectron 2, for detecting all wrist fractures, including scaphoid, on 1,917 radiographs from 637 patients [17]. The system achieved an overall sensitivity of 83%, a specificity of 96%, and an AUC of 0.840, with scaphoid-specific sensitivity of 84%, comparable to inter-rater reliability among radiologists (80%). Notably, AI sensitivity (83%) exceeded that of initial radiologist reads (76%), and the combined AI-assisted approach achieved 88% sensitivity, demonstrating that AI augmentation improved diagnostic performance over either approach alone. This represents an important example of commercially deployed AI systems achieving practical clinical utility in real-world settings.

This represented the highest rate of occult fracture detection reported in the literature to date and a dramatic improvement over human expert performance. The authors hypothesized that segmentation-based training with explicit fracture contour annotation may better capture subtle radiographic features associated with occult injuries compared to classification or bounding box approaches. This finding is particularly clinically significant because occult fracture detection is precisely where human observers struggle most and where missed diagnoses lead to the most severe long-term complications, including non-union, avascular necrosis, and chronic wrist dysfunction.

Benchmark Meta-Analysis, Prognostic Modelling and Healing Prediction

The field's aggregated performance was synthesized in the meta-analysis by Kraus et al., pooling seven CNN-based studies encompassing 3,373 images [18]. The pooled analysis demonstrated sensitivity of 0.80 (95% CI: 0.75-0.84), specificity of 0.89 (95% CI: 0.82-0.94), and AUC of 0.88 across heterogeneous studies, with individual study AUCs ranging from 0.77 to 0.96. This provided high diagnostic accuracy benchmarks and offers a useful reference for assessing the performance of individual studies and tracking the field's progress over time. The analysis also revealed that AI performance varies by anatomical area, with carpal bone sensitivity of 56% (41% when excluding scaphoid), indicating room for improvement in detecting fractures of smaller carpal bones.

Two studies extended DL beyond fracture detection into healing prediction and prognostic assessment. Su et al. used YOLO (segmentation), DenseNet169, and ResNet50 (classification) on 346 patients to evaluate follow-up radiographs. Working with 346 patients, they trained separate models for surgical treatment recommendations (668 images: 334 surgery, 334 no surgery) and prognosis classification (87 images: 29 each of union, non-union, and avascular necrosis). The surgical recommendation model using DenseNet169 achieved 80% accuracy and AUC of 0.86, while the prognosis classification model using ResNet50 achieved 91% accuracy and AUC of 0.96 [19]. Despite the small prognosis dataset, the model demonstrated that radiographic texture features can reliably distinguish healing states, establishing the feasibility of automated union detection, a task traditionally limited by significant interobserver variability among radiologists and clinicians.

Tümen et al., building on this concept, trained a custom CNN using YOLOv4 for segmentation, followed by a TensorFlow CNN with transfer learning on postoperative radiographs from 346 patients with established scaphoid non-unions [20]. The model predicted surgical reconstruction outcomes with 93.63% accuracy, far exceeding logistic regression performance (66.3%). This was the first study to correlate AI-derived image features with actual surgical outcomes, identifying smoking and intraoperative avascular necrosis as significant factors. The study shifted focus from static diagnosis to longitudinal prognostication [20]. These studies highlight AI's emerging role as a quantitative biomarker of bone healing.

Clinical Translation

Transfer-learning architectures (DenseNet, ResNet, EfficientNet) achieve high performance with modest datasets and generalize well across sites. Segmentation-first models provide enhanced sensitivity for occult fractures (41% versus 0-50% for classification approaches) and superior interpretability through fracture contour visualization. By outlining fracture contours rather than relying on bounding boxes, they generate intuitive heatmaps that integrate seamlessly with picture archiving and communication systems (PACS), improving clinician trust and supporting explainable AI standards. Multi-view fusion consistently improves recall by 10-15 percentage points. By combining anteroposterior and lateral projections, these models more closely replicate radiological practice and improve diagnostic reliability. Reader-assistance studies confirm AI’s role as a complement rather than a replacement for human expertise.

The prospective validation study by Lee et al. demonstrated that AI support improved novice radiologists' diagnostic accuracy by 10-14 AUC percentage points, demonstrating tangible clinical value [14]. Collectively, these findings underscore AI’s potential to enhance decision-making, particularly in emergency or resource-limited settings, while highlighting the importance of continued optimization for smooth integration into clinical workflows. The high negative predictive value (97.2%) achieved by Yoon et al.’s two-stage model makes it particularly suitable for ruling out fractures in settings where advanced imaging is not readily available [9].

Study limitations and future directions

Significant heterogeneity across the included studies limited quantitative synthesis. The investigations differed in model architecture, dataset size (87-12,990 images), validation strategy (single-centre versus multicentre), and definitions of key entities such as occult fractures. Reporting of performance metrics was inconsistent, ranging from simple accuracy to full diagnostic indices, hindering direct comparison and meta-analysis. Most studies were retrospective and single-centre, introducing potential selection bias and limiting generalisability to other imaging systems and populations. Reference standards for ground truth varied widely, including CT, MRI, cone-beam CT, or clinical follow-up, with some studies reporting results without clear specification, creating possible verification bias.

Future research should prioritize prospective multicentre validation, standardized reference definitions, and adoption of reporting frameworks (CONSORT-AI, CLAIM). Healing prediction models require larger datasets with longer follow-up and clinical covariates. Cost-effectiveness analyses weighing implementation costs against gains in accuracy, efficiency, and patient outcomes remain lacking but essential for widespread adoption.

## Conclusions

DL models show encouraging potential for assisting in the detection of scaphoid fractures on radiographs, particularly in augmenting diagnostic confidence and reducing variability. Although several studies demonstrate high accuracy, the current evidence base remains limited, and true equivalence to expert radiologist performance has not yet been established. Early exploratory work in fracture-healing prediction is promising, but these findings are preliminary and should be interpreted cautiously until replicated in larger, more diverse cohorts. Advances in segmentation algorithms, transfer learning, and multimodal imaging integration highlight the technical feasibility of AI-assisted interpretation; however, their clinical applicability depends on rigorous validation. At present, the literature is dominated by single-centre datasets, retrospective designs, and heterogeneous reporting standards. Future research must prioritise multicentre external validation, prospective implementation studies, transparent explainability frameworks, and adherence to regulatory and ethical requirements. While AI may eventually play a supportive role in improving diagnostic pathways for scaphoid fractures, current evidence supports its potential rather than its readiness for widespread clinical adoption. Continued refinement and validation will determine whether these technologies can meaningfully enhance workflow efficiency, reduce missed diagnoses, and support patient-centred outcomes in routine practice.
